# New generalized systems of nonlinear ordered variational inclusions involving ⊕ operator in real ordered Hilbert spaces

**DOI:** 10.1186/s13660-018-1846-0

**Published:** 2018-09-21

**Authors:** Mohd. Sarfaraz, Kottakkaran Sooppy Nisar, Ahmed Morsy, Md. Kalimuddin Ahmad

**Affiliations:** 1 Department of Mathematics, Aligarh Muslim University, Aligarh, India; 2 Department of Mathematics, College of Arts and Sciences at Wadi Al-dawasir, Prince Sattam Bin Abdulaziz University, Riyadh Region, Kingdom of Saudi Arabia

**Keywords:** 47J05, 47J20, Algorithm, Weak-ARD mapping, Nonlinear ordered variational inclusions, System, Ordered Hilbert space

## Abstract

This manuscript deals with two general systems of nonlinear ordered variational inclusion problems. We also construct some new iterative algorithms for finding approximation solutions to the general systems of nonlinear ordered variational inclusions and prove the convergence of the sequences obtained by the schemes. The results presented in the manuscript are new and improve some well-known results in the literature.

## Introduction

A lot of work has been added into the theory of variational inequalities since its seed was planted by Lions et al. [24]. On account of its wide applications in physics and applied sciences etc., the classical variational inequalities have been extensively studied by many researchers in different ways [1, 4, 5, 7–10].

A useful and important generalization of variational inequality problem is variational inclusion problem which was introduced and studied by Hasounni et al. [16]. Furthermore, they proposed a perturbed iterative algorithm for solving the variational inclusion problem.

Fang et al. [12] introduced and studied *H*-monotone operators, which was used to design a resolvent operator and to prove its Lipschitz continuity. Furthermore, they also introduced a class of variational inclusions in Hilbert space. Fang et al. [13] additionally presented another class of generalized monotone operators, called $(H,\eta)$-monotone operators, which generalize different classes of maximal monotone, maximal *η*-monotone and *H*-monotone operators.

Recently, Lan et al. [17] presented another idea of $(A,\eta )$-accretive mappings, which generalized the current monotone or accretive operators, and concentrated a few properties of mappings. They examined a class of variational inclusions using the resolvent operator related with $(A,\eta)$-accretive mappings.

Amann [6] studied the number of fixed points for a continuous operator $A:[x,y] \rightarrow[x,y]$ on a bounded order interval $[x,y] \subset\mathcal{E}$, an ordered Banach space. The nonlinear mapping fixed point theory and applications have been widely studied in ordered Banach spaces [4, 14, 15]. In this manner, it is essential that summed up nonlinear ordered variational inclusions (ordered equation) are contemplated.

Plenty of research concerned with the ordered equations and ordered variational inequalities in ordered Banach spaces has been done by Li et al.; see [21, 23]. Many problems concerning ordered variational inclusions are answered by the resolvent technique linked with RME set-valued mappings [19], $(\alpha, \lambda)$-NODM set-valued mapping [20], $(\gamma_{G}, \lambda)$-weak RRD mapping [2] and $(\alpha, \lambda)$-weak ANODD set-valued map with strongly comparison mapping *A* [21] and many more see; e.g., [3, 22, 25, 26, 29] and the references therein.

In this work, we make use of the resolvent operator approach for the approximation solvability of solutions of implicit system of generalized nonlinear ordered variational inclusions in real ordered Hilbert spaces.

## Preliminaries

In this part, we present some basic notions and results for the building up the manuscript.

Allow $\mathcal{E}$ to be a real ordered Hilbert space endowed with a norm $\|\cdot\|$, and an inner product $\langle\cdot,\cdot\rangle$, *d* be a metric induced by the norm $\|\cdot\|$, $CB(\mathcal{E})$ be a collection of all closed and bounded subsets of $\mathcal{E}$ and $D(\cdot, \cdot)$ be a Hausdorff metric on $CB(\mathcal{E})$ defined as

$$D(M,N)= \max\Bigl\{ \sup_{x \in M} d(x, N), \sup _{y \in N} d(M, y) \Bigr\} , $$

where $M, N \in CB(\mathcal{E})$, $d(x, N)= \inf_{y \in N}d(x, y)$ and $d(M, y)= \inf_{x \in M}d(x, y)$.

### Definition 2.1

Let $\mathfrak{C}$ be a nonvoid closed, convex subset of $\mathcal{E}$. Then $\mathfrak{C}$ is called a cone if

- $x\in\mathfrak{C}$ and $\kappa>0$, $\kappa x \in\mathfrak{C}$;
- *x* and $-x\in\mathfrak{C}$, then $x=\Theta$.

### Definition 2.2

([11])

A cone $\mathfrak{C}$ is said to be normal iff there exists $\lambda _{N_{C}} >0$ with $0\leq x \leq y$ implying $\|x\| \leq\lambda_{N_{C}} \|y\|$, where $\lambda_{N_{C}}$ is called a normal constant of $\mathfrak{C}$.

### Definition 2.3

A relation ≤ defined as $x \leq y$ iff $y-x \in\mathfrak{C}$ for $x, y \in\mathcal{E}$ is known as a partial order relation expounded by $\mathfrak{C}$ in $\mathcal{E}$; then ($\mathcal{E}, \leq$) is called a real ordered Hilbert space.

### Definition 2.4

([27])

Members $x, y \in\mathcal{E}$ having the relation $x \leq y$ (or $y \leq x$) are called comparable with each other.

### Definition 2.5

([27])

For arbitrary elements $x, y \in\mathcal{E}$, $\operatorname{lub}\{x, y\}$ and $\operatorname{glb}\{ x, y\}$ mean the least upper bound and the greatest upper bound of the set $\{x, y\}$. Suppose $\operatorname{lub}\{x, y\}$ and $\operatorname{glb}\{x, y\}$ exist; some binary relations are defined as follows:

- $x \vee y = \operatorname{lub}\{x, y\}$;
- $x \wedge y = \operatorname{glb}\{x, y\}$;
- $x \oplus y = (x-y) \vee(y-x)$;
- $x \odot y = (x-y) \wedge(y-x)$.

The operations ∨, ∧, ⊕ and ⊙ are called **OR, AND, XOR** and **XNOR** operations, respectively.

### Proposition 1

([11])

*For any positive integer* *n*, *if* $x \propto y_{n}$ *and* $y_{n} \rightarrow y^{\ast}$ ($n \rightarrow\infty$), *then* $x \propto y^{\ast}$.

### Proposition 2

([11, 20])

*Let* $\textbf{XOR}, \textbf{XNOR}$ *be two operations on* $\mathcal{E}$. *Then the following hold*:

- $x \odot x = 0, x \odot y= y \odot x= -(x \oplus y)=-(y \oplus x)$;
- $(\lambda x) \oplus(\lambda y)= |\lambda| (x \oplus y)$;
- $x \odot0 \leq0$, *if* $x \propto0$;
- $0 \leq x \oplus y $, *if* $x \propto y$;
- *if* $x \propto y$, *then* $x \oplus y= 0$ *if and only if* $x=y$;
- $(x+y) \odot(u+v) \geq(x \odot u) + (y \odot v)$;
- $(x+y) \odot(u+v) \geq(x \odot v) + (y \odot u)$;
- $(\alpha x \oplus\beta x)= |\alpha- \beta|x$, *if* $x \propto0$, $\forall x, y, u, v \in\mathcal{E}$ *and* $\alpha, \beta, \lambda\in\mathbb{R}$.

### Proposition 3

([11])

*Let* $\mathfrak{C}$ *be a normal cone in* $\mathcal{E}$ *with normal constant* $\lambda_{N_{C}}$, *then*, *for each* $x, y \in\mathcal{E}$, *the following hold*:

- $\|0+0\|= \|0\|=0$;
- $\|x \vee y\| \leq\|x\|\vee\|y\| \leq\|x\|+ \|y\|$;
- $\|x \oplus y\|\leq\|x-y\|\leq\lambda_{N_{C}}\|x \oplus y\|$;
- *if* $x \propto y$, *then* $\|x \oplus y\|= \|x-y\|$.

### Definition 2.6

([20])

Let $A:\mathcal{E} \rightarrow\mathcal{E}$ to be a single-valued map.

- *A* is called a *δ*-order non-extended map, if there is a positive constant $\delta> 0$ such that
  $$\delta(x \oplus y) \leq A(x) \oplus A(y) \quad \mbox{for all } x, y \in \mathcal{E}; $$
- *A* is called a strongly comparison map, if it is a comparison map and $A(x) \propto A(y)$ iff $x \propto y$, for all $x, y \in\mathcal{E}$.

### Definition 2.7

([2])

A single-valued map $A: \mathcal{E}\rightarrow\mathcal{E}$ is termed a *β*-ordered compression, if it is comparison map and

$$A(x)\oplus A(y) \leq\beta(x\oplus y), \quad \mbox{for } 0 < \beta< 1. $$

### Definition 2.8

([18])

A map $A:\mathcal{E}\times\mathcal{E}\rightarrow\mathcal{E}$ is called $(\alpha_{1},\alpha_{2})$-restricted-accretive map, if it is a comparison and ∃ constants $0 \leq\alpha_{1}, \alpha_{2} \leq1$ such that

$$\bigl(A(x,\cdot)+I(x)\bigr)\oplus\bigl(A(y,\cdot)+I(y)\bigr) \leq \alpha_{1}\bigl(A(x,\cdot) \oplus A(y,\cdot)\bigr) + \alpha_{2}(x \oplus y),\quad \mbox{for all } x, y \in \mathcal{E} $$

where *I* is the identity map on $\mathcal{E}$.

### Lemma 2.1

([28])

*Let* $\theta\in(0, 1)$ *be a constant*. *Then the function* $f(\lambda )=1-\lambda+\lambda\theta$ *for* $\lambda\in[0,1]$ *is nonnegative and strictly decreases and* $f(\lambda) \in[0, 1]$. *Furthermore*, *if* $\lambda\neq0$, *then* $f(\lambda) \in(0,1)$.

### Lemma 2.2

([30])

*Assume that* $\{a_{n}\}$ *and* $\{b_{n}\}$ *be two sequences of nonnegative real numbers such that*

$$a_{n+1} \leq\theta a_{n} + b_{n}, $$

*where* $\theta\in(0,1)$ *and* $\lim_{n\rightarrow\infty}b_{n} = 0$. *Then* $\lim_{n\rightarrow\infty} a_{n}=0$.

## Ordered weak-ARD mapping in ordered Hilbert spaces

### Definition 3.1

Let $A: \mathcal{E}\rightarrow\mathcal{E}$ be a strong comparison and *β*-ordered compression mapping and $M:\mathcal{E}\rightarrow CB(\mathcal{E})$ be a set-valued mapping. Then

- *M* is said to be a comparison mapping, if for any $v_{x} \in M(x)$, $x \propto v_{x}$ and if $x \propto y$, then, for any $v_{x} \in M(x)$ and any $v_{y} \in M(y)$, $v_{x} \propto v_{y}$, for all $x, y \in\mathcal{E}$;
- a comparison mapping *M* is said to be ordered rectangular, if for each $x, y \in\mathcal{E}$, $v_{x} \in M(x)$ and $v_{y} \in M(y)$ such that
  $$\bigl\langle v_{x}\odot v_{y}, -(x\oplus y)\bigr\rangle =0; $$
- a comparison mapping *M* is said to be a *γ*-ordered rectangular with respect to *A*, if there exists a constant $\gamma_{A} > 0$ for any $x, y \in\mathcal{E}$, there exist $v_{x}\in M(A(x))$ and $v_{y}\in M(A(y))$ such that
  $$\bigl\langle v_{x}\odot v_{y}, -\bigl(A(x)\oplus A(y) \bigr)\bigr\rangle \geq\gamma_{A} \bigl\Vert A(x)\oplus A(y) \bigr\Vert ^{2}, $$
  holds, where $v_{x} $ and $v_{y}$ are said to be $\gamma _{A}$-elements, respectively;
- *M* is said to be a weak comparison mapping with respect to *A*, if, for any $x, y \in\mathcal{E}$, $x \propto y$, there exist $v_{x}\in M(A(x))$ and $v_{y}\in M(A(y))$ such that $x \propto v_{x}$, $y \propto v_{y}$, where $v_{x}$ and $v_{y}$ are said to be weak comparison elements, respectively;
- *M* is said to be a *λ*-weak ordered different comparison mapping with respect to *A*, if there exists a constant $\lambda> 0$ such that, for any $x, y \in\mathcal{E}$, there exist $v_{x}\in M(A(x))$ and $v_{y}\in M(A(y))$, $\lambda(v_{x}-v_{y})\propto (x-y)$ holds, where $v_{x}$ and $v_{y}$ are said to be *λ*-elements, respectively;
- a weak comparison mapping *M* is said to be a $(\gamma_{A}, \lambda)$-weak ARD mapping with respect to *A*, if *M* is a $\gamma_{A}$-ordered rectangular and *λ*-weak ordered different comparison mapping with respect to *A* and $(A+\lambda M)(\mathcal{E})= \mathcal{E}$, for $\lambda> 0$ and there exist $v_{x}\in M(A(x))$ and $v_{y}\in M(A(y))$ such that $v_{x}$ and $v_{y}$ are $(\gamma_{A}, \lambda)$-elements, respectively.

### Definition 3.2

A set-valued mapping $A:\mathcal{E} \rightarrow CB(\mathcal{E})$ is said to be $\delta_{A}$-Lipschitz continuous, if for each $x, y \in\mathcal{E}, x \propto y$, there exists a constant $\delta_{A}$ such that

$$D\bigl(A(x), A(y)\bigr) \leq\delta_{A} \Vert x \oplus y \Vert , \quad \forall x, y \in\mathcal{E}. $$

### Definition 3.3

Let $M:\mathcal{E}\rightarrow CB(\mathcal{E})$ be a set-valued mapping, $A:\mathcal{E}\rightarrow\mathcal{E}$ be a single-valued mapping and $I:\mathcal{E}\rightarrow\mathcal{E}$ be an identity mapping. Then a weak comparison mapping *M* is said to be a $(\gamma^{\prime}, \lambda)$-weak-ARD mapping with respect to $(I-A)$, if *M* is a $\gamma^{\prime}$-ordered rectangular and *λ*-weak ordered different comparison mapping with respect to $(I-A)$ and $[(I-A)+\lambda M](\mathcal{E})= \mathcal {E}$, for $\lambda> 0$ and there exist $v_{x} \in M((I-A)(x))$ and $v_{y} \in M((I-A)(y))$ such that $v_{x}$ and $v_{y}$ are called $(\gamma^{\prime}, \lambda)$-elements, respectively.

### Definition 3.4

Let $\mathfrak{C}$ be a normal cone with normal constant $\lambda _{N_{C}}$ and $M:\mathcal{E}\rightarrow CB(\mathcal{E})$ be a weak-ARD set-valued mapping. Let $I:\mathcal{E}\rightarrow\mathcal {E}$ be the identity mapping and $A:\mathcal{E}\rightarrow\mathcal{E}$ be a set-valued mapping and $A:\mathcal{E}\rightarrow\mathcal{E}$ be a single-valued mapping. The relaxed resolvent operator $R_{M,\lambda}^{(I-A)}: \mathcal {E}\rightarrow\mathcal{E}$ associated with *I*, *A* and *M* is defined by

1 $$ R_{M,\lambda}^{(I-A)}(x)= \bigl[(I-A)+\lambda M \bigr]^{-1}(x), \quad \forall x \in\mathcal{E} \mbox{ and } \lambda>0. $$

The relaxed resolvent operator defined by (1) is single-valued, a comparison mapping and Lipschitz continuous.

### Proposition 4

([2])

*Let* $A:\mathcal{E}\rightarrow\mathcal{E}$ *be a* *β*-*ordered compression mapping and* $M:\mathcal{E}\rightarrow CB(\mathcal{E})$ *be the set*-*valued ordered rectangular mapping*. *Then the resolvent* $R_{M,\lambda}^{(I-A)}: \mathcal{E}\rightarrow\mathcal{E}$ *is single*-*valued*, *for all* $\lambda > 0$.

### Proposition 5

([2])

*Let* $M:\mathcal{E}\rightarrow CB(\mathcal{E})$ *be a* $(\gamma_{A}, \lambda)$-*weak*-*ARD set*-*valued mapping with respect to* $R_{M,\lambda}^{(I-A)}$. *Let* $A:\mathcal{E}\rightarrow\mathcal{E}$ *be a strongly comparison mapping with respect to* $R_{M,\lambda}^{(I-A)}$ *and* $I: \mathcal{E}\rightarrow\mathcal{E}$ *be the identity mapping*. *Then the resolvent operator* $R_{M,\lambda}^{(I-A)}: \mathcal{E}\rightarrow\mathcal{E}$ *is a comparison mapping*.

### Proposition 6

([2])

*Let* $M:\mathcal{E}\rightarrow CB(\mathcal{E})$ *be a* $(\gamma_{A}, \lambda)$-*weak*-*ARD set*-*valued mapping with respect to* $R_{M,\lambda}^{(I-A)}$. *Let* $A:\mathcal{E}\rightarrow\mathcal{E}$ *be a strongly comparison and* *β*-*ordered compression mapping with respect to* $R_{M,\lambda}^{(I-A)}$ *with condition* $\lambda\gamma_{A}> \beta+1$. *Then the following condition survives*:

$$ \bigl\Vert R_{M,\lambda}^{(I-A)}(x) \oplus R_{M,\lambda}^{(I-A)}(y) \bigr\Vert \leq \biggl(\frac{1}{\lambda\gamma_{A}-\beta-1} \biggr) \Vert x \oplus y \Vert . $$

## Formulation of the problems

Let $F_{i}: \mathcal{E}_{1}\times\mathcal{E}_{2}\times\cdots\times \mathcal{E}_{m}\rightarrow\mathcal{E}_{i}$, $A_{i}: \mathcal{E}_{i} \rightarrow\mathcal{E}_{i}$ and $g_{i}: \mathcal{E}_{i}\rightarrow \mathcal{E}_{i}$ to be single-valued mappings, for $i, j= 1,2,3,\ldots, m$. Let $U_{ij}: \mathcal{E}_{j} \rightarrow CB(\mathcal{E}_{j})$ be a set-valued map and $M_{i}: \mathcal{E}_{i}\rightarrow CB(\mathcal {E}_{i})$ be set-valued weak-ARD mapping. Then we have the problem:

Find $(x_{1}^{\ast},x_{2}^{\ast}, \ldots, x_{m}^{\ast}) \in\mathcal {E}_{1}\times\mathcal{E}_{2}\times\cdots\times\mathcal{E}_{m}$ and $u_{ij}^{\ast} \in U_{ij}(x_{j}^{\ast})$, for $i, j= 1,2,3,\ldots m$, such that

2 $$ 0 \in\rho_{i} F_{i} \bigl(u_{i1}^{\ast},u_{i2}^{\ast}, \ldots, u_{im}^{\ast} \bigr) \oplus\lambda_{i} M_{i} \bigl(g_{i} \bigl(x_{i}^{\ast} \bigr) \bigr), $$

where $\rho_{i}$ and $\lambda_{i}$ are given positive constants. Problem (2) is called a generalized set-valued system of nonlinear ordered variational inclusions problem for weak-ARD mappings.

If $U_{ij}= T_{ij}$ is a single-valued mapping, then problem (2) becomes:

Find $x_{j} \in\mathcal{E}_{j}$, such that

3 $$ 0 \in\rho_{i} F_{i} \bigl(T_{i1}x_{1}^{\ast },T_{i2}x_{2}^{\ast}, \ldots, T_{im}x_{m}^{\ast} \bigr) \oplus \lambda_{i} M_{i} \bigl(g_{i} \bigl(x_{i}^{\ast} \bigr) \bigr). $$

This problem is known as a generalized system of nonlinear ordered variational inclusions problem involving weak-ARD mappings.

### Remark

Here, we discuss special cases for our problem (2), which was encountered by Li et al.

Case 1.: For $i, j=1$, $\rho_{i} = 1$, $\lambda_{i} = 1$ and $U_{ij}= g_{i} =I$, then problem (2) is reduced to finding $x \in\mathcal{E}_{1}$ such that
  4 $$ 0 \in F_{1}(x)\oplus M_{1}(x). $$
  This problem was considered by Li et al. [23] and coined a general nonlinear mixed-order quasi-variational inclusion (GNMOQVI) involving the ⊕ operator in an ordered Banach space.
Case 2.: If $F=0$ (zero mapping), then problem (4) is reduced to finding $x \in\mathcal{E}$ such that
  5 $$ 0 \in M(x). $$
  This problem were considered by Li for ordered RME set-valued mappings [19] and $(\alpha, \lambda)$-NODM set-valued mappings [20].

### Lemma 4.1

*Let* $(x_{1}^{\ast},x_{2}^{\ast}, \ldots, x_{m}^{\ast}) \in\mathcal {E}_{1}\times\mathcal{E}_{2}\times\cdots\times\mathcal{E}_{m}$ *and* $u_{ij}^{\ast} \in U_{ij}(x_{j}^{\ast})$ *for* $i, j= 1,2,3,\ldots, m$. *Then* $(x_{1}^{\ast},x_{2}^{\ast}, \ldots, x_{m}^{\ast}, u_{11}^{\ast}, u_{12}^{\ast}, \ldots, u_{1m}^{\ast}, \ldots, u_{m1}^{\ast},u_{m2}^{\ast}, \ldots, u_{mm}^{\ast})$ *is a solution of problem* (2) *if and only if it satisfies*

6 $$ g_{i} \bigl(x_{i}^{\ast} \bigr) = J_{\lambda_{i}, M_{i}}^{I_{i}-A_{i}} \bigl[(I_{i}-A_{i}) \bigl(g_{i} \bigl(x_{i}^{\ast} \bigr) \bigr) + \rho_{i} F_{i} \bigl(u_{i1}^{\ast},u_{i2}^{\ast}, \ldots, u_{im}^{\ast} \bigr) \bigr], $$

*where* $J_{\lambda_{i}, M_{i}}^{I_{i}-A_{i}}(x)= [(I_{i}-A_{i})+ \lambda _{i} M_{i}]^{-1}(x)$ *and* $\rho_{i}, \lambda_{i} > 0$ *for* $i=1, 2,\ldots , m$.

### Proof

The proof follows from the definition of the relaxed resolvent operator. □

## Design of the algorithms

### Remark

If we choose $\lambda= 1$ and $U_{ij}= T_{ij}$ for $i, j=1, 2,\ldots, m$, is single-valued operator, then Algorithm 1 reduces to Algorithm 2 for problem (3).

**Algorithm 1:**
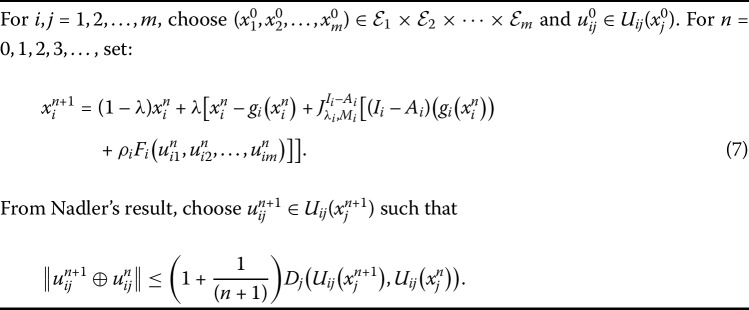
**for the problem (** **2** **):**

**Algorithm 2:**
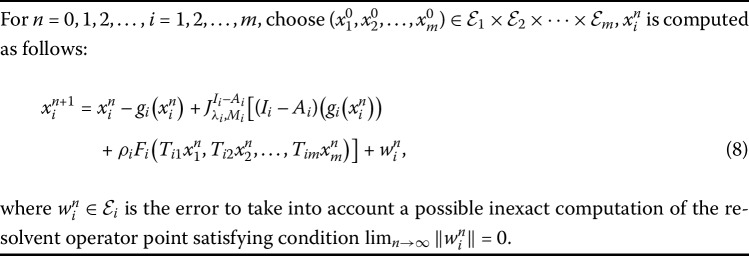
**for the problem (** **3** **):**

## Main results

### Theorem 6.1

*Let* $A_{i}: \mathcal{E}_{i}\rightarrow\mathcal{E}_{i}$, $g_{i}: \mathcal{E}_{i}\rightarrow\mathcal{E}_{i}$ *and* $F_{i}: \mathcal {E}_{1}\times\mathcal{E}_{2}\times\cdots\times\mathcal {E}_{m}\rightarrow\mathcal{E}_{i}$ *be the single*-*valued mappings such that* $A_{i}$ *be* $\lambda_{A_{i}}$-*ordered compression mapping*, $g_{i}$ *be* $\lambda_{g_{i}}$-*ordered compression*, $(\alpha_{1}^{i},\alpha _{2}^{i})$-*ordered restricted*-*accretive mapping and* $F_{i}$ *be* $\lambda_{ij}$-*ordered compression mapping with respect to the* *jth argument*. *Let* $U_{ij}: \mathcal{E}_{j} \rightarrow CB(\mathcal{E}_{j})$ *be a* $D_{i}$-$\delta_{ij}$-*ordered Lipschitz continuous set*-*valued mapping*. *Let* $M_{i}: \mathcal{E}_{i}\rightarrow CB(\mathcal{E}_{i})$ *be a* $(\gamma_{A_{i}}, \lambda_{i})$-*weak rectangular different compression mapping with respect to* $A_{i}$ *and if* $x_{i} \propto y_{i}$, $J_{\lambda_{i}, M_{i}}^{I_{i}-A_{i}}(x_{i}) \propto J_{\lambda_{i}, M_{i}}^{I_{i}-A_{i}}(y_{i})$ *and for all* $\lambda_{i}$, $\rho_{i} > 0$, *then the following condition holds*:

9 $$ \theta_{j}= \Biggl\{ \alpha_{1}^{j}+ \alpha_{2}^{j}\lambda_{g_{j}}+ L_{j}( \lambda_{g_{j}}+\lambda_{A_{j}}\lambda_{g_{j}}) + \sum _{i\neq j, i=1}^{m}L_{i} \rho_{i}\lambda_{F_{ij}}\delta_{D_{ij}} \Biggr\} < 1, $$

*for all* $j=1,2,3, \ldots,m$, *which in turn*, *implies that problem* (2) *admits a solution* $(x_{1}^{\ast},x_{2}^{\ast},\ldots, x_{m}^{\ast }, u_{11}^{\ast},u_{12}^{\ast},\ldots,u_{1m}^{\ast},\ldots ,u_{m1}^{\ast},\ldots,u_{mm}^{\ast})$, *where* $(x_{1}^{\ast},x_{2}^{\ast },\ldots,x_{m}^{\ast}) \in\mathcal{E}_{1}\times\mathcal{E}_{2}\times \cdots\times\mathcal{E}_{m}$ *and* $u_{ij}^{\ast} \in U_{ij}(x_{j}^{\ast})$. *Moreover*, *iterative sequences* $\{x_{j}^{n}\}$ *and* $\{u_{ij}^{n}\}$ *generated by Algorithm *1, *converge strongly to* $x_{j}^{\ast}$ *and* $u_{ij}^{\ast}$, *for* $i, j=1, 2,\ldots,m$, *respectively*.

### Proof

Using Algorithm 1 and Proposition 2, for $i=1, 2,\ldots, m$, we have

10 $$\begin{aligned} x_{i}^{n+1} \oplus x_{i}^{n}={}& \bigl((1-\lambda) x_{i}^{n} + \lambda \bigl[x_{i}^{n}-g_{i} \bigl(x_{i}^{n} \bigr)+J_{\lambda_{i}, M_{i}}^{I_{i}-A_{i}} \bigl[(I_{i}-A_{i}) \bigl(g_{i} \bigl(x_{i}^{n} \bigr) \bigr) \\ &{}+\rho_{i} F_{i} \bigl(u_{i1}^{n},u_{i2}^{n}, \ldots, u_{im}^{n} \bigr) \bigr] \bigr] \bigr)\oplus \bigl((1- \lambda) x_{i}^{n-1} \\ &{}+\lambda \bigl[x_{i}^{n-1}-g_{i} \bigl(x_{i}^{n-1} \bigr)+J_{\lambda_{i}, M_{i}}^{I_{i}-A_{i}} \bigl[(I_{i}-A_{i}) \bigl(g_{i} \bigl(x_{i}^{n-1} \bigr) \bigr) \\ &{}+\rho_{i} F_{i} \bigl(u_{i1}^{n-1},u_{i2}^{n-1}, \ldots, u_{im}^{n-1} \bigr) \bigr] \bigr] \bigr) \\ \leq{}&(1-\lambda) \bigl(x_{i}^{n}\oplus x_{i}^{n-1} \bigr)+\lambda \bigl[ \bigl(x_{i}^{n}-g_{i} \bigl(x_{i}^{n} \bigr) \bigr)\oplus \bigl(x_{i}^{n-1}-g_{i} \bigl(x_{i}^{n-1} \bigr) \bigr) \bigr] \\ &{}+\lambda \bigl\{ J_{\lambda_{i}, M_{i}}^{I_{i}-A_{i}} \bigl[(I_{i}-A_{i}) \bigl(g_{i} \bigl(x_{i}^{n} \bigr) \bigr) + \rho_{i} F_{i} \bigl(u_{i1}^{n},u_{i2}^{n}, \ldots, u_{im}^{n} \bigr) \bigr] \\ &{}\oplus J_{\lambda_{i}, M_{i}}^{I_{i}-A_{i}} \bigl[(I_{i}-A_{i}) \bigl(g_{i} \bigl(x_{i}^{n-1} \bigr) \bigr) + \rho_{i} F_{i} \bigl(u_{i1}^{n-1},u_{i2}^{n-1}, \ldots, u_{im}^{n-1} \bigr) \bigr] \bigr\} \\ \leq{}&(1-\lambda) \bigl(x_{i}^{n}\oplus x_{i}^{n-1} \bigr)+\lambda(\alpha_{1}^{i} \bigl(x_{i}^{n}\oplus x_{i}^{n-1} \bigr)+ \alpha_{2}^{i} \bigl(g_{i} \bigl(x_{i}^{n} \bigr)\oplus g_{i} \bigl(x_{i}^{n-1} \bigr) \bigr) \\ &{}+\lambda \bigl\{ J_{\lambda_{i}, M_{i}}^{I_{i}-A_{i}} \bigl[(I_{i}-A_{i}) \bigl(g_{i} \bigl(x_{i}^{n} \bigr) \bigr) + \rho_{i} F_{i} \bigl(u_{i1}^{n},u_{i2}^{n}, \ldots, u_{im}^{n} \bigr) \bigr] \\ &{}\oplus J_{\lambda_{i}, M_{i}}^{I_{i}-A_{i}} \bigl[(I_{i}-A_{i}) \bigl(g_{i} \bigl(x_{i}^{n-1} \bigr) \bigr) + \rho_{i} F_{i} \bigl(u_{i1}^{n-1},u_{i2}^{n-1}, \ldots, u_{im}^{n-1} \bigr) \bigr] \bigr\} \\ \leq{}&(1-\lambda) \bigl(x_{i}^{n}\oplus x_{i}^{n-1} \bigr)+\lambda \bigl(\alpha_{1}^{i}+ \alpha_{2}^{i}\lambda_{g_{i}} \bigr) \bigl(x_{i}^{n}\oplus x_{i}^{n-1} \bigr) \\ &{}+\lambda \bigl\{ J_{\lambda_{i}, M_{i}}^{I_{i}-A_{i}} \bigl[(I_{i}-A_{i}) \bigl(g_{i} \bigl(x_{i}^{n} \bigr) \bigr) + \rho_{i} F_{i} \bigl(u_{i1}^{n},u_{i2}^{n}, \ldots, u_{im}^{n} \bigr) \bigr] \\ &{}\oplus J_{\lambda_{i}, M_{i}}^{I_{i}-A_{i}} \bigl[(I_{i}-A_{i}) \bigl(g_{i} \bigl(x_{i}^{n-1} \bigr) \bigr) + \rho_{i} F_{i} \bigl(u_{i1}^{n-1},u_{i2}^{n-1}, \ldots, u_{im}^{n-1} \bigr) \bigr] \bigr\} . \end{aligned}$$

Using Definition 2.2, Proposition 6 and Eq. (10), we get

11 $$\begin{aligned} \bigl\Vert x_{i}^{n+1} \oplus x_{i}^{n} \bigr\Vert \leq{}&\lambda_{N_{C}} \bigl[(1- \lambda)+\lambda \bigl(\alpha_{1}^{i}+\lambda_{g_{i}} \alpha_{2}^{i} \bigr) \bigr] \bigl\Vert x_{i}^{n} \oplus x_{i}^{n-1} \bigr\Vert \\ &{}+\lambda\lambda_{N_{C}}L_{i} \bigl\Vert \bigl[(I_{i}-A_{i}) \bigl(g_{i} \bigl(x_{i}^{n} \bigr) \bigr)+\rho_{i} F_{i} \bigl(u_{i1}^{n},u_{i2}^{n}, \ldots, u_{im}^{n} \bigr) \bigr] \\ &{}\oplus \bigl[(I_{i}-A_{i}) \bigl(g_{i} \bigl(x_{i}^{n-1} \bigr) \bigr)+\rho_{i} F_{i} \bigl(u_{i1}^{n-1},u_{i2}^{n-1}, \ldots, u_{im}^{n-1} \bigr) \bigr] \bigr\Vert \\ \leq{}&\lambda_{N_{C}} \bigl[1-\lambda \bigl(1- \bigl(\alpha_{1}^{i}+ \lambda_{g_{i}}\alpha_{2}^{i} \bigr) \bigr) \bigr] \bigl\Vert x_{i}^{n}\oplus x_{i}^{n-1} \bigr\Vert \\ &{}+\lambda\lambda_{N_{C}}L_{i} \bigl[ \bigl\Vert (I_{i}-A_{i}) \bigl(g_{i} \bigl(x_{i}^{n} \bigr) \bigr)\oplus(I_{i}-A_{i}) \bigl(g_{i} \bigl(x_{i}^{n-1} \bigr) \bigr) \bigr\Vert \bigr] \\ &{}+\lambda\lambda_{N_{C}}L_{i}\rho_{i} \bigl\Vert F_{i} \bigl(u_{i1}^{n},u_{i2}^{n}, \ldots, u_{im}^{n} \bigr)\oplus F_{i} \bigl(u_{i1}^{n-1},u_{i2}^{n-1},\ldots, u_{im}^{n-1} \bigr) \bigr\Vert \\ \leq{}&\lambda_{N_{C}} \bigl[1-\lambda \bigl(1- \bigl(\alpha_{1}^{i}+ \lambda_{g_{i}}\alpha_{2}^{i} \bigr) \bigr) \bigr] \bigl\Vert x_{i}^{n}\oplus x_{i}^{n-1} \bigr\Vert \\ &{}+\lambda\lambda_{N_{C}}L_{i} \bigl[ \bigl\Vert \bigl(g_{i} \bigl(x_{i}^{n} \bigr)\oplus g_{i} \bigl(x_{i}^{n} \bigr) \bigr) \bigr\Vert + \bigl\Vert A_{i} \bigl(g_{i} \bigl(x_{i}^{n} \bigr) \bigr)\oplus A_{i} \bigl(g_{i} \bigl(x_{i}^{n-1} \bigr) \bigr) \bigr\Vert \bigr] \\ &{}+\lambda\lambda_{N_{C}}L_{i}\rho_{i} \bigl[ \bigl\Vert F_{i} \bigl(u_{i1}^{n},u_{i2}^{n}, \ldots, u_{im}^{n} \bigr)\oplus F_{i} \bigl(u_{i1}^{n-1},u_{i2}^{n-1},\ldots, u_{im}^{n-1} \bigr) \bigr\Vert \bigr] \\ \leq{}&\lambda_{N_{C}} \bigl[1-\lambda \bigl(1- \bigl(\alpha_{1}^{i}+ \lambda_{g_{i}}\alpha_{2}^{i} \bigr) \bigr) \bigr] \bigl\Vert x_{i}^{n}\oplus x_{i}^{n-1} \bigr\Vert \\ &{}+\lambda\lambda_{N_{C}}L_{i} \bigl[( \lambda_{g_{i}}+\lambda_{A_{i}}\lambda_{g_{i}}) \bigl\Vert x_{i}^{n}\oplus x_{i}^{n-1} \bigr\Vert \bigr] \\ &{}+\lambda\lambda_{N_{C}}L_{i}\rho_{i} \bigl[ \bigl\Vert F_{i} \bigl(u_{i1}^{n},u_{i2}^{n}, \ldots, u_{im}^{n} \bigr)\oplus F_{i} \bigl(u_{i1}^{n-1},u_{i2}^{n-1},\ldots, u_{im}^{n-1} \bigr) \bigr\Vert \bigr]. \end{aligned}$$

Now, from Eq. (11), we compute

12 $$\begin{aligned} & \bigl\Vert F_{i} \bigl(u_{i1}^{n},u_{i2}^{n}, \ldots,u_{ii-1}^{n},u_{ii}^{n},u_{ii+1}^{n}, \ldots,u_{im}^{n} \bigr)\oplus F_{i} \bigl(u_{i1}^{n-1},u_{i2}^{n-1},\ldots, u_{ii-1}^{n-1},u_{ii}^{n-1}, u_{ii+1}^{n-1},\ldots,u_{im}^{n-1} \bigr) \bigr\Vert \\ &\quad \leq \bigl\Vert F_{i} \bigl(u_{i1}^{n},u_{i2}^{n}, \ldots,u_{ii-1}^{n},u_{ii}^{n},u_{ii+1}^{n}, \ldots,u_{im}^{n} \bigr) \\ &\qquad{} \oplus F_{i} \bigl(u_{i1}^{n-1},u_{i2}^{n}, \ldots,u_{ii-1}^{n},u_{ii}^{n},u_{ii+1}^{n}, \ldots,u_{im}^{n} \bigr) \bigr\Vert \\ &\qquad{}+ \bigl\Vert F_{i} \bigl(u_{i1}^{n-1},u_{i2}^{n}, \ldots,u_{ii-1}^{n},u_{ii}^{n},u_{ii+1}^{n}, \ldots,u_{im}^{n} \bigr) \\ &\qquad{}\oplus F_{i} \bigl(u_{i1}^{n-1},u_{i2}^{n-1}, \ldots,u_{ii-1}^{n-1},u_{ii}^{n-1},u_{ii+1}^{n-1}, \ldots,u_{im}^{n-1} \bigr) \bigr\Vert +\cdots \\ &\qquad{}+ \bigl\Vert F_{i} \bigl(u_{i1}^{n-1},u_{i2}^{n-1}, \ldots,u_{ii-1}^{n-1},u_{ii}^{n-1},u_{im-1}^{n-1},u_{im}^{n} \bigr) \\ &\qquad{} \oplus F_{i} \bigl(u_{i1}^{n-1},u_{i2}^{n-1}, \ldots,u_{ii-1}^{n-1},u_{ii}^{n-1},u_{ii+1}^{n-1}, \ldots,u_{im}^{n-1} \bigr) \bigr\Vert . \end{aligned}$$

By the definition of $F_{i}$ as a $\lambda_{F_{ij}}$-ordered compression map with respect to the *j*th argument, we have

13 $$\begin{aligned} &\bigl\Vert F_{i} \bigl(u_{i1}^{n},u_{i2}^{n}, \ldots,u_{ii-1}^{n},u_{ii}^{n},u_{ii+1}^{n}, \ldots,u_{im}^{n} \bigr)\oplus F_{i} \bigl(u_{i1}^{n-1},u_{i2}^{n-1}, \ldots,u_{ii-1}^{n-1},u_{ii}^{n-1}, u_{ii+1}^{n-1},\ldots,u_{im}^{n-1} \bigr) \bigr\Vert \\ &\quad \leq\lambda_{F_{i1}} \bigl\Vert u_{i1}^{n} \oplus u_{i1}^{n-1} \bigr\Vert +\lambda_{F_{i2}} \bigl\Vert u_{i2}^{n}\oplus u_{i2}^{n-1} \bigr\Vert +\cdots+\lambda_{F_{im}} \bigl\Vert u_{im}^{n} \oplus u_{im}^{n-1} \bigr\Vert \\ &\quad = \sum_{i\neq j, j=1}^{m} \lambda_{F_{ij}} \bigl\Vert u_{ij}^{n}\oplus u_{ij}^{n-1} \bigr\Vert \\ &\quad \leq\sum_{i\neq j, j=1}^{m} \lambda_{F_{ij}} \biggl(1+\frac{1}{(n+1)} \biggr) D_{j} \bigl(U_{ij} \bigl(x_{j}^{n} \bigr), U_{ij} \bigl(x_{j}^{n-1} \bigr) \bigr) \\ &\quad \leq \biggl(1+\frac{1}{(n+1)} \biggr) \sum_{i\neq j, j=1}^{m} \lambda_{F_{ij}} \delta_{D_{ij}} \bigl\Vert x_{j}^{n} \oplus x_{j}^{n-1} \bigr\Vert . \end{aligned}$$

Using Proposition 6 and Eq. (13) in Eq. (11), we obtain

$$\begin{aligned} \bigl\Vert x_{i}^{n+1} \oplus x_{i}^{n} \bigr\Vert ={}& \bigl\Vert x_{i}^{n+1} - x_{i}^{n} \bigr\Vert \\ \leq{}&\lambda_{N_{C}} \bigl[1-\lambda \bigl(1- \bigl(\alpha_{1}^{i}+ \lambda_{g_{i}}\alpha_{2}^{i} \bigr) \bigr) \bigr] \bigl\Vert x_{i}^{n}- x_{i}^{n-1} \bigr\Vert \\ &{}+\lambda\lambda_{N_{C}}L_{i} \bigl[( \lambda_{g_{i}}+\lambda_{A_{i}}\lambda_{g_{i}}) \bigl\Vert x_{i}^{n}- x_{i}^{n-1} \bigr\Vert \bigr] \\ &{}+\lambda\lambda_{N_{C}}L_{i}\rho_{i} \biggl(1+\frac{1}{(n+1)} \biggr) \sum_{i\neq j, j=1}^{m} \lambda_{F_{ij}} \delta_{D_{ij}} \bigl\Vert x_{j}^{n}- x_{j}^{n-1} \bigr\Vert \\ \leq{}& \bigl\{ \lambda_{N_{C}} \bigl[1-\lambda \bigl(1- \bigl( \alpha_{1}^{i}+\alpha_{2}^{i} \lambda_{g_{i}} \bigr) \bigr) \bigr]+ \lambda\lambda_{N_{C}}L_{i}( \lambda_{g_{i}}+\lambda_{A_{i}}\lambda_{g_{i}}) \bigr\} \bigl\Vert x_{i}^{n}-x_{i}^{n-1} \bigr\Vert \\ &{}+\lambda\lambda_{N_{C}}L_{i}\rho_{i} \biggl(1+\frac{1}{(n+1)} \biggr) \sum_{i\neq j, j=1}^{m} \lambda_{F_{ij}}\delta_{D_{ij}} \bigl\Vert x_{j}^{n}- x_{j}^{n-1} \bigr\Vert , \end{aligned}$$

which implies that

14 $$\begin{aligned} &\sum_{j=1}^{m} \bigl\Vert x_{j}^{n+1} - x_{j}^{n} \bigr\Vert \\ &\quad = \sum_{i=1}^{m} \bigl\Vert x_{i}^{n+1}-x_{i}^{n} \bigr\Vert \\ &\quad \leq\sum_{i=1}^{m} \Biggl\{ \bigl[ \lambda_{N_{C}} \bigl[1-\lambda \bigl(1- \bigl(\alpha_{1}^{i}+ \alpha_{2}^{i}\lambda_{g_{i}} \bigr) \bigr) \bigr]+ \lambda\lambda_{N_{C}}L_{i}(\lambda_{g_{i}}+ \lambda_{A_{i}}\lambda_{g_{i}}) \bigr] \bigl\Vert x_{i}^{n} - x_{i}^{n-1} \bigr\Vert \\ &\qquad{}+\lambda\lambda_{N_{C}} \biggl(1+\frac{1}{(n+1)} \biggr) \sum _{i\neq j, j=1}^{m} L_{j} \rho_{j}\lambda_{F_{ij}}\delta_{D_{ij}} \bigl\Vert x_{j}^{n}- x_{j}^{n-1} \bigr\Vert \Biggr\} \\ &\quad = \sum_{i=1}^{m} \lambda_{N_{C}} \bigl[1-\lambda \bigl\{ 1- \bigl(\alpha_{1}^{i}+ \lambda_{g_{i}}\alpha_{2}^{i} \bigr) +L_{i}( \lambda_{g_{i}}+\lambda_{A_{i}} \lambda_{g_{i}}) \bigr\} \bigr] \bigl\Vert x_{i}^{n}-x_{i}^{n-1} \bigr\Vert \\ &\qquad{}+\lambda\lambda_{N_{C}} \biggl(1+\frac{1}{(n+1)} \biggr)\sum _{i=1}^{m}\sum_{i\neq j, j=1}^{m}L_{j} \rho_{j}\lambda_{F_{ij}}\delta_{D_{ij}} \bigl\Vert x_{j}^{n}-x_{j}^{n-1} \bigr\Vert \\ &\quad = \sum_{j=1}^{m}\lambda_{N_{C}} \bigl[1-\lambda \bigl\{ 1- \bigl(\alpha_{1}^{j}+ \alpha_{2}^{j}\lambda_{g_{j}} \bigr)+ L_{j}( \lambda_{g_{j}}+\lambda_{A_{j}} \lambda_{g_{j}}) \bigr\} \bigr] \bigl\Vert x_{j}^{n}-x_{j}^{n-1} \bigr\Vert \\ &\qquad{}+\lambda_{N_{C}}\lambda \biggl(1+\frac{1}{(n+1)} \biggr)\sum _{j=1}^{m}\sum_{i\neq j, i=1}^{m}L_{j} \rho_{j}\lambda_{F_{ij}}\delta_{D_{ij}} \bigl\Vert x_{j}^{n}-x_{j}^{n-1} \bigr\Vert \\ &\quad = \sum_{j=1}^{m}\lambda_{N_{C}} \Biggl[1-\lambda+\lambda \Biggl\{ \alpha_{1}^{j}+ \alpha_{2}^{j}\lambda_{g_{j}}+ L_{j}( \lambda_{g_{j}}+\lambda_{A_{j}}\lambda_{g_{j}}) \\ &\qquad{}+ \biggl(1+\frac{1}{(n+1)} \biggr)\sum_{i\neq j, i=1}^{m}L_{i} \rho_{i}\lambda_{F_{ij}}\delta_{D_{ij}} \Biggr\} \Biggr] \bigl\Vert x_{j}^{n}-x_{j}^{n-1} \bigr\Vert \\ &\quad = \sum_{j=1}^{m}\lambda_{N_{C}} \bigl(1-\lambda+\lambda\theta_{j}^{n} \bigr) \bigl\Vert x_{j}^{n}-x_{j}^{n-1} \bigr\Vert \\ &\quad \leq\lambda_{N_{C}} f_{n}(\lambda) \sum _{j=1}^{m} \bigl\Vert x_{j}^{n}-x_{j}^{n-1} \bigr\Vert , \end{aligned}$$

where

$$\theta_{j}^{n}= \Biggl\{ \alpha_{1}^{j}+ \alpha_{2}^{j}\lambda_{g_{j}}+ L_{j}( \lambda_{g_{j}}+\lambda_{A_{j}}\lambda_{g_{j}}) + \biggl(1+ \frac{1}{(n+1)} \biggr)\sum_{i\neq j, i=1}^{m}L_{i} \rho_{i}\lambda _{F_{ij}}\delta_{D_{ij}} \Biggr\} < 1 $$

and

$$f_{n}(\lambda)=\max_{1\leq j \leq m}\bigl\{ 1-\lambda+\lambda \theta_{j}^{n}\bigr\} . $$

From Eq. (14), we know that the sequence $\{\theta_{j}^{n}\}$ is monotonic decreasing and $\theta_{j}^{n}\rightarrow\theta_{j}$ as $n\rightarrow\infty$. Thus, $f(\lambda)= \lim_{n\rightarrow\infty}f_{n}(\lambda)=\max_{1\leq j\leq m}\{1-\lambda+\lambda\theta_{j}\}$. Since $0 < \theta_{j} < 1$ for $j=1, 2,\ldots, m$. We get $\theta= \max_{1\leq j \leq m}\{\theta_{j}\} \in(0, 1)$. By Lemma 2.1, we have $f(\lambda)= 1- \lambda+ \lambda\theta\in (0, 1)$, from Eq. (14), it follows that $\{x_{j}^{n}\}$ is a Cauchy sequence and there exists $x_{j}^{\ast} \in\mathcal{E}_{j}$ such that $x_{j}^{n} \rightarrow x_{j}^{\ast}$ as $n \rightarrow\infty$ for $j = 1, 2,\ldots, m$. Next, we show that $u_{ij}^{n} \rightarrow u_{ij}^{\ast} \in U_{ij}(x_{j}^{\ast})$ as $n \rightarrow\infty$ for $i, j= 1, 2,\ldots, m$. It follows from Eq. (13) that the $\{u_{ij}^{n}\}$ are also Cauchy sequences. Hence, there exists $u_{ij}^{\ast} \in\mathcal{E}_{j}$ such that $u_{ij}^{n} \rightarrow u_{ij}^{\ast}$ as $n\rightarrow\infty$ for $i, j= 1, 2,\ldots, m$. Furthermore,

$$\begin{aligned} d \bigl(u_{ij}^{\ast}, U_{ij} \bigl(x_{j}^{\ast} \bigr) \bigr) =& \inf \bigl\{ \bigl\Vert u_{ij}^{\ast} \oplus t \bigr\Vert : t \in U_{ij} \bigl(x_{j}^{\ast} \bigr) \bigr\} \\ \leq& \bigl\Vert u_{ij}^{\ast} \oplus u_{ij}^{n} \bigr\Vert + d \bigl(u_{ij}^{n}, U_{ij} \bigl(x_{j}^{\ast} \bigr) \bigr) \\ \leq& \bigl\Vert u_{ij}^{\ast} \oplus u_{ij}^{n} \bigr\Vert + d \bigl(U_{ij} \bigl(x_{j}^{n} \bigr), U_{ij} \bigl(x_{j}^{\ast} \bigr) \bigr) \\ \leq& \bigl\Vert u_{ij}^{\ast} \oplus u_{ij}^{n} \bigr\Vert +\delta_{D_{ij}} \bigl\Vert x_{j}^{\ast} \oplus x_{j}^{n} \bigr\Vert \\ \leq& \bigl\Vert u_{ij}^{\ast}- u_{ij}^{n} \bigr\Vert +\delta_{D_{ij}} \bigl\Vert x_{j}^{\ast}-x_{j}^{n} \bigr\Vert \rightarrow0\quad (n\rightarrow\infty). \end{aligned}$$

Since $U_{ij}(x_{j}^{\ast})$ is closed for $i, j=1, 2,\ldots, m$, we have $u_{ij}^{\ast} \in U_{ij}(x_{j}^{\ast})$ for $i, j=1, 2,\ldots, m$. By using continuity $(x_{1}^{\ast}, x_{2}^{\ast}, \ldots, x_{m}^{\ast}) \in\mathcal{E}_{1}\times\mathcal{E}_{2}\times\cdots\times\mathcal {E}_{m}$ and $u_{ij}^{\ast} \in U_{ij}(x_{j}^{\ast})$ for $i, j=1, 2,\ldots, m$ satisfy Eq. (6) and so by Lemma 4.1, problem (2) has a solution $(x_{1}^{\ast},x_{2}^{\ast}, \ldots, x_{m}^{\ast}, u_{11}^{\ast}, u_{12}^{\ast}, \ldots, u_{1m}^{\ast}, \ldots, u_{m1}^{\ast},u_{m2}^{\ast}, \ldots, u_{mm}^{\ast})$, where $u_{ij}^{\ast}\in U_{ij}(x_{j}^{\ast})$ for $i, j=1, 2,\ldots, m$ and $(x_{1}^{\ast}, x_{2}^{\ast},\ldots, x_{m}^{\ast}) \in\mathcal {E}_{1}\times\mathcal{E}_{2}\times\cdots\times\mathcal{E}_{m}$. This completes the proof. □

### Theorem 6.2

*Suppose that* $A_{i}, g_{i}$ *and* $M_{i}$ *are the same as in Theorem* 6.1 *for* $i=1, 2,\ldots,m$. *Let* $T_{ij}: \mathcal{E}_{j}\rightarrow\mathcal{E}_{j}$ *be* $\gamma _{ij}$-*Lipschitz continuous and* $F_{i}:\mathcal{E}_{1}\times \mathcal{E}_{2}\times\cdots\times\mathcal{E}_{m}\rightarrow\mathcal {E}_{i}$ *be* $\lambda_{F_{ij}}$-*ordered compression mapping with respect to the* *jth argument*. *Let there be constants* $\lambda_{j} >0$, *for* $j=1,2,\ldots,m$ *such that*

$$ \theta_{j}= \Biggl[\lambda_{N_{C}} \bigl\{ \bigl( \alpha_{1}^{j}+\alpha_{2}^{j} \lambda_{g_{i}} \bigr)+L_{j}(\lambda_{g_{j}}+ \lambda_{A_{j}}\lambda_{g_{j}}) \bigr\} + \lambda_{N_{C}} \sum_{i\neq j, i=1}^{m} L_{i} \rho_{i}\lambda_{F_{ij}}\gamma_{ij} \Biggr]< 1. $$

*Then problem* (3) *has a unique solution* $(x_{1}^{\ast},x_{2}^{\ast },\ldots,x_{m}^{\ast})\in\mathcal{E}_{1}\times\mathcal{E}_{2}\times \cdots\times\mathcal{E}_{m}$. *Moreover*, *the iterative sequence* $\{ x_{j}^{n}\}$ *generated by Algorithm* 2 *converges strongly to* $x_{j}^{\ast }$ *for* $j=1,2,\ldots,m$.

### Proof

Let us define a norm $\|\cdot\|_{\ast}$ on the product space $\mathcal {E}_{1}\times\mathcal{E}_{2}\times\cdots\times\mathcal{E}_{m}$ by

15 $$ \bigl\Vert (x_{1},x_{2}, \ldots,x_{m}) \bigr\Vert _{\ast}= \sum _{i=1}^{m} \Vert x_{j} \Vert ,\quad \forall (x_{1},x_{2},\ldots,x_{m})\in \mathcal{E}_{1}\times\mathcal{E}_{2}\times\cdots\times \mathcal{E}_{m}. $$

Then it can easily be seen that $(\mathcal{E}_{1}\times\mathcal {E}_{2}\times\cdots\times\mathcal{E}_{m}, \|\cdot\|_{\ast})$ is a Banach space.

Setting

$$\begin{aligned} y_{i} =& x_{i}-g_{i}(x_{i})+ J_{\lambda_{i}, M_{i}}^{I_{i}-A_{i}} \bigl[(I_{i}-A_{i}) \bigl(g_{i}(x_{i}) \bigr) \\ & {}+\rho_{i} F_{i}(T_{i1}x_{1},\ldots,T_{ii-1}x_{i-1}, T_{ii}x_{i},T_{ii+1}x_{i+1}, \ldots,T_{im}x_{m}) \bigr]. \end{aligned}$$

Define a mapping $Q:\mathcal{E}_{1}\times\mathcal{E}_{2}\times\cdots \times\mathcal{E}_{m} \rightarrow\mathcal{E}_{1}\times\mathcal {E}_{2}\times\cdots\times\mathcal{E}_{m}$ as

$$ Q(x_{1},x_{2},\ldots,x_{m})=(y_{1},y_{2}, \ldots,y_{m}),\quad \forall (x_{1},x_{2}, \ldots,x_{m})\in\mathcal{E}_{1}\times\mathcal{E}_{2} \times\cdots\times\mathcal{E}_{m}. $$

For any $(x_{1}^{1},x_{2}^{1},\ldots,x_{m}^{1})$, $(x_{1}^{2},x_{2}^{2},\ldots,x_{m}^{2})\in\mathcal{E}_{1}\times\mathcal {E}_{2}\times\cdots\times\mathcal{E}_{m}$ we have

16 $$\begin{aligned} & \bigl\Vert Q \bigl(x_{1}^{1},x_{2}^{1}, \ldots,x_{m}^{1} \bigr)\oplus Q \bigl(x_{1}^{2},x_{2}^{2}, \ldots,x_{m}^{2} \bigr) \bigr\Vert _{\ast} \\ &\quad \leq \bigl\Vert Q \bigl(x_{1}^{1},x_{2}^{1}, \ldots,x_{m}^{1} \bigr)-Q \bigl(x_{1}^{2},x_{2}^{2}, \ldots,x_{m}^{2} \bigr) \bigr\Vert _{\ast} \\ &\quad \leq \bigl\Vert \bigl(y_{1}^{1},y_{2}^{1}, \ldots,y_{m}^{1} \bigr)- \bigl(y_{1}^{2},y_{2}^{2}, \ldots,y_{m}^{2} \bigr) \bigr\Vert _{\ast} \\ &\quad \leq \sum_{i=1}^{m} \bigl\Vert y_{i}^{1}- y_{i}^{2} \bigr\Vert . \end{aligned}$$

First of all, we have to calculate $(y_{i}^{1} \oplus y_{i}^{2})$ as follows:

$$\begin{aligned} \bigl(y_{i}^{1} \oplus y_{i}^{2} \bigr) =& \bigl(x_{i}^{1}-g_{i} \bigl(x_{i}^{1} \bigr)+ J_{\lambda_{i}, M_{i}}^{I_{i}-A_{i}} \bigl[(I_{i}-A_{i}) \bigl(g_{i} \bigl(x_{i}^{1} \bigr) \bigr) \\ &{}+\rho_{i} F_{i} \bigl(T_{i1}x_{1}^{1}, \ldots,T_{ii-1}x_{i-1}^{1},T_{ii}x_{i}^{1},T_{ii+1}x_{i+1}^{1}, \ldots,T_{im}x_{m}^{1} \bigr) \bigr] \bigr) \\ &{}\oplus \bigl(x_{i}^{2}-g_{i} \bigl(x_{i}^{2} \bigr)+ J_{\lambda_{i}, M_{i}}^{I_{i}-A_{i}} \bigl[(I_{i}-A_{i}) \bigl(g_{i} \bigl(x_{i}^{2} \bigr) \bigr) \\ &{}+\rho_{i} F_{i} \bigl(T_{i1}x_{1}^{2}, \ldots,T_{ii-1}x_{i-1}^{2},T_{ii}x_{i}^{2},T_{ii+1}x_{i+1}^{2}, \ldots,T_{im}x_{m}^{2} \bigr) \bigr] \bigr) \\ =& \bigl( \bigl(x_{i}^{1}-g_{i} \bigl(x_{i}^{1} \bigr) \bigr)\oplus \bigl(x_{i}^{2}-g_{i} \bigl(x_{i}^{2} \bigr) \bigr) \bigr) + \bigl(J_{\lambda_{i}, M_{i}}^{I_{i}-A_{i}} \bigl[(I_{i}-A_{i}) \bigl(g_{i} \bigl(x_{i}^{1} \bigr) \bigr) \\ &{}+\rho_{i} F_{i} \bigl(T_{i1}x_{1}^{1}, \ldots,T_{ii-1}x_{i-1}^{1},T_{ii}x_{i}^{1},T_{ii+1}x_{i+1}^{1}, \ldots,T_{im}x_{m}^{1} \bigr) \bigr] \\ &{}\oplus J_{\lambda_{i}, M_{i}}^{I_{i}-A_{i}} \bigl[(I_{i}-A_{i}) \bigl(g_{i} \bigl(x_{i}^{2} \bigr) \bigr) \\ &{}+\rho_{i} F_{i} \bigl(T_{i1}x_{1}^{2}, \ldots,T_{ii-1}x_{i-1}^{2},T_{ii}x_{i}^{2},T_{ii+1}x_{i+1}^{2}, \ldots,T_{im}x_{m}^{2} \bigr) \bigr] \bigr). \end{aligned}$$

From Definition 2.2 and Proposition 3, we have

17 $$\begin{aligned} \bigl\Vert y_{i}^{1} \oplus y_{i}^{2} \bigr\Vert \leq& \bigl\Vert y_{i}^{1} - y_{i}^{2} \bigr\Vert \\ \leq& \lambda_{N_{C}} \bigl\Vert \bigl\{ \bigl( \bigl(x_{i}^{1}-g_{i} \bigl(x_{i}^{1} \bigr) \bigr) \\ &{}\oplus \bigl(x_{i}^{2}-g_{i} \bigl(x_{i}^{2} \bigr) \bigr) \bigr) + \bigl(J_{\lambda_{i}, M_{i}}^{I_{i}-A_{i}} \bigl[(I_{i}-A_{i}) \bigl(g_{i} \bigl(x_{i}^{1} \bigr) \bigr) \\ &{}+\rho_{i} F_{i} \bigl(T_{i1}x_{1}^{1}, \ldots,T_{ii-1}x_{i-1}^{1},T_{ii}x_{i}^{1},T_{ii+1}x_{i+1}^{1}, \ldots,T_{im}x_{m}^{1} \bigr) \bigr] \\ &{}\oplus J_{\lambda_{i}, M_{i}}^{I_{i}-A_{i}} \bigl[(I_{i}-A_{i}) \bigl(g_{i} \bigl(x_{i}^{2} \bigr) \bigr) \\ &{}+\rho_{i} F_{i} \bigl(T_{i1}x_{1}^{2}, \ldots,T_{ii-1}x_{i-1}^{2},T_{ii}x_{i}^{2},T_{ii+1}x_{i+1}^{2}, \ldots,T_{im}x_{m}^{2} \bigr) \bigr] \bigr) \bigr\} \bigr\Vert \\ \leq& \lambda_{N_{C}} \bigl\{ \alpha_{1}^{j} \bigl\Vert x_{i}^{1}- x_{i}^{2} \bigr\Vert +\alpha_{2}^{j}\lambda_{g_{i}} \bigl\Vert x_{i}^{1}- x_{i}^{2} \bigr\Vert \bigr\} \\ &{}+\lambda_{N_{C}} \bigl\Vert \bigl(J_{\lambda_{i}, M_{i}}^{I_{i}-A_{i}} \bigl[(I_{i}-A_{i}) \bigl(g_{i} \bigl(x_{i}^{1} \bigr) \bigr)+\rho_{i} F_{i} \bigl(T_{i1}x_{1}^{1}, \ldots,T_{ii-1}x_{i-1}^{1}, \\ &{}T_{ii}x_{i}^{1},T_{ii+1}x_{i+1}^{1}, \ldots,T_{im}x_{m}^{1} \bigr) \bigr]\oplus J_{\lambda_{i}, M_{i}}^{I_{i}-A_{i}} \bigl[(I_{i}-A_{i}) \bigl(g_{i} \bigl(x_{i}^{2} \bigr) \bigr) \\ &{}+\rho_{i} F_{i} \bigl(T_{i1}x_{1}^{2}, \ldots,T_{ii-1}x_{i-1}^{2},T_{ii}x_{i}^{2},T_{ii+1}x_{i+1}^{2}, \ldots,T_{im}x_{m}^{2} \bigr) \bigr] \bigr) \bigr\Vert \\ \leq& \lambda_{N_{C}} \bigl(\alpha_{1}^{j}+ \alpha_{2}^{j}\lambda_{g_{i}} \bigr) \bigl\Vert x_{i}^{1}- x_{i}^{2} \bigr\Vert + \lambda_{N_{C}} \bigl\Vert \bigl(J_{\lambda_{i}, M_{i}}^{I_{i}-A_{i}} \bigl[(I_{i}-A_{i}) \bigl(g_{i} \bigl(x_{i}^{1} \bigr) \bigr) \\ &{}+\rho_{i} F_{i} \bigl(T_{i1}x_{1}^{1}, \ldots,T_{ii-1}x_{i-1}^{1},T_{ii}x_{i}^{1},T_{ii+1}x_{i+1}^{1}, \ldots,T_{im}x_{m}^{1} \bigr) \bigr] \\ &{}\oplus J_{\lambda_{i}, M_{i}}^{I_{i}-A_{i}} \bigl[(I_{i}-A_{i}) \bigl(g_{i} \bigl(x_{i}^{2} \bigr) \bigr) \\ &{}+\rho_{i} F_{i} \bigl(T_{i1}x_{1}^{2}, \ldots,T_{ii-1}x_{i-1}^{2},T_{ii}x_{i}^{2},T_{ii+1}x_{i+1}^{2}, \ldots,T_{im}x_{m}^{2} \bigr) \bigr] \bigr) \bigr\Vert . \end{aligned}$$

Further, we calculate

18 $$\begin{aligned} & \bigl\Vert J_{\lambda_{i}, M_{i}}^{I_{i}-A_{i}} \bigl[(I_{i}-A_{i}) \bigl(g_{i} \bigl(x_{i}^{1} \bigr) \bigr)+\rho_{i}F_{i} \bigl(T_{i1}x_{1}^{1},\ldots ,T_{ii-1}x_{i-1}^{1},T_{ii}x_{i}^{1},T_{ii+1}x_{i+1}^{1}, \ldots, T_{im}x_{m}^{1} \bigr) \bigr] \\ &\qquad {}\oplus J_{\lambda_{i}, M_{i}}^{I_{i}-A_{i}} \bigl[(I_{i}-A_{i}) \bigl(g_{i} \bigl(x_{i}^{2} \bigr) \bigr) + \rho_{i}F_{i} \bigl(T_{i1}x_{1}^{2}, \ldots,T_{ii-1}x_{i-1}^{2},T_{ii}x_{i}^{2},T_{ii+1}x_{i+1}^{2}, \ldots,T_{im}x_{m}^{2} \bigr) \bigr] \bigr\Vert \\ &\quad \leq L_{i} \bigl[ \bigl\Vert \bigl\{ (I_{i}-A_{i}) \bigl(g_{i} \bigl(x_{i}^{1} \bigr) \bigr)\oplus (I_{i}-A_{i}) \bigl(g_{i} \bigl(x_{i}^{2} \bigr) \bigr) \bigr\} \bigr\Vert \\ &\qquad{}+\rho_{i} \bigl\Vert F_{i} \bigl(T_{i1}x_{1}^{1}, \ldots,T_{ii-1}x_{i-1}^{1},T_{ii}x_{i}^{1},T_{ii+1}x_{i+1}^{1}, \ldots,T_{im}x_{m}^{1} \bigr) \\ &\qquad{}\oplus F_{i} \bigl(T_{i1}x_{1}^{2}, \ldots,T_{ii-1}x_{i-1}^{2},T_{ii}x_{i}^{2},T_{ii+1}x_{i+1}^{2}, \ldots,T_{im}x_{m}^{2} \bigr) \bigr\Vert \bigr] \\ &\quad \leq L_{i} \bigl[ \bigl\Vert \bigl(g_{i} \bigl(x_{i}^{1} \bigr)\oplus g_{i} \bigl(x_{i}^{2} \bigr) \bigr)+ \bigl(A_{i} \bigl(g_{i} \bigl(x_{i}^{1} \bigr) \bigr)\oplus A_{i} \bigl(g_{i} \bigl(x_{i}^{2} \bigr) \bigr) \bigr) \bigr\Vert \\ &\qquad{}+\rho_{i} \bigl\Vert F_{i} \bigl(T_{i1}x_{1}^{1}, \ldots,T_{ii-1}x_{i-1}^{1},T_{ii}x_{i}^{1},T_{ii+1}x_{i+1}^{1}, \ldots,T_{im}x_{m}^{1} \bigr) \\ &\qquad {} \oplus F_{i} \bigl(T_{i1}x_{1}^{2}, \ldots,T_{ii-1}x_{i-1}^{2},T_{ii}x_{i}^{2},T_{ii+1}x_{i+1}^{2}, \ldots,T_{im}x_{m}^{2} \bigr) \bigr\Vert \bigr] \\ &\quad \leq L_{i} \bigl[ \bigl\{ \lambda_{g_{i}} \bigl\Vert x_{i}^{1}\oplus x_{i}^{2} \bigr\Vert +\lambda_{A_{i}}\lambda_{g_{i}} \bigl\Vert x_{i}^{1} \oplus x_{i}^{2} \bigr\Vert \bigr\} \\ &\qquad{}+\rho_{i} \bigl\Vert F_{i} \bigl(T_{i1}x_{1}^{1}, \ldots,T_{ii-1}x_{i-1}^{1},T_{ii}x_{i}^{1},T_{ii+1}x_{i+1}^{1}, \ldots,T_{im}x_{m}^{1} \bigr) \\ &\qquad {}\oplus F_{i} \bigl(T_{i1}x_{1}^{2}, \ldots,T_{ii-1}x_{i-1}^{2},T_{ii}x_{i}^{2},T_{ii+1}x_{i+1}^{2}, \ldots,T_{im}x_{m}^{2} \bigr) \bigr\Vert \bigr] \\ &\quad \leq L_{i} \bigl[(\lambda_{g_{i}}+\lambda_{A_{i}} \lambda_{g_{i}}) \bigl\Vert x_{i}^{1}\oplus x_{i}^{2} \bigr\Vert \\ &\qquad{}+\rho_{i} \bigl\Vert F_{i} \bigl(T_{i1}x_{1}^{1}, \ldots,T_{ii-1}x_{i-1}^{1},T_{ii}x_{i}^{1},T_{ii+1}x_{i+1}^{1}, \ldots,T_{im}x_{m}^{1} \bigr) \\ &\qquad{} F_{i} \bigl(T_{i1}x_{1}^{2}, \ldots,T_{ii-1}x_{i-1}^{2},T_{ii}x_{i}^{2},T_{ii+1}x_{i+1}^{2}, \ldots,T_{im}x_{m}^{2} \bigr) \bigr\Vert \bigr]. \end{aligned}$$

Now we calculate the inner part estimate of the above expression with the help of the properties of the $F_{i}$-operator for $i=1, 2,\ldots ,m$. We have

19 $$\begin{aligned} & \bigl\Vert F_{i} \bigl(T_{i1}x_{1}^{1}, \ldots,T_{ii-1}x_{i-1}^{1},T_{ii}x_{i}^{1},T_{ii+1}x_{i+1}^{1}, \ldots,T_{im}x_{m}^{1} \bigr) \\ &\qquad {} \oplus F_{i} \bigl(T_{i1}x_{1}^{2}, \ldots,T_{ii-1}x_{i-1}^{2},T_{ii}x_{i}^{2}, T_{ii+1}x_{i+1}^{2},\ldots,T_{im}x_{m}^{2} \bigr) \bigr\Vert \\ &\quad = \bigl\Vert F_{i} \bigl(T_{i1}x_{1}^{1}, \ldots,T_{ii-1}x_{i-1}^{1},T_{ii}x_{i}^{1},T_{ii+1}x_{i+1}^{1}, \ldots,T_{im}x_{m}^{1} \bigr)\oplus F_{i} \bigl(T_{i1}x_{1}^{2}, \ldots,T_{ii-1}x_{i-1}^{1}, \\ &\qquad{} T_{ii}x_{i}^{1},T_{ii+1}x_{i+1}^{1}, \ldots,T_{im}x_{m}^{1} \bigr) \oplus F_{i} \bigl(T_{i1}x_{1}^{2}, \ldots,T_{ii-1}x_{i-1}^{1},T_{ii}x_{i}^{1}, \\ &\qquad{} T_{ii+1}x_{i+1}^{1},\ldots,T_{im}x_{m}^{1} \bigr)\oplus F_{i} \bigl(T_{i1}x_{1}^{2}, \ldots, T_{ii-1}x_{i-1}^{2},T_{ii}x_{i}^{2},T_{ii+1}x_{i+1}^{2}, \ldots,T_{im}x_{m}^{2} \bigr) \bigr\Vert \\ &\quad \leq \bigl\Vert F_{i} \bigl(T_{i1}x_{1}^{1}, \ldots,T_{ii-1}x_{i-1}^{1},T_{ii}x_{i}^{1},T_{ii+1}x_{i+1}^{1}, \ldots,T_{im}x_{m}^{1} \bigr) \\ &\qquad{}\oplus F_{i} \bigl(T_{i1}x_{1}^{2}, \ldots,T_{ii-1}x_{i-1}^{1},T_{ii}x_{i}^{1},T_{ii+1}x_{i+1}^{1}, \ldots,T_{im}x_{m}^{1} \bigr) \bigr\Vert +\cdots \\ &\qquad{}+ \bigl\Vert F_{i} \bigl(T_{i1}x_{1}^{2}, \ldots,T_{ii-1}x_{i-1}^{1},T_{ii}x_{i}^{1},T_{ii+1}x_{i+1}^{1}, \ldots,T_{im}x_{m}^{1} \bigr) \\ &\qquad{}\oplus F_{i} \bigl(T_{i1}x_{1}^{2}, \ldots,T_{ii-1}x_{i-1}^{2},T_{ii}x_{i}^{1},T_{ii+1}x_{i+1}^{1}, \ldots,T_{im}x_{m}^{1} \bigr) \bigr\Vert \\ &\qquad{}+ \bigl\Vert F_{i} \bigl(T_{i1}x_{1}^{2}, \ldots,T_{ii-1}x_{i-1}^{2},T_{ii}x_{i}^{1},T_{ii+1}x_{i+1}^{1}, \ldots,T_{im}x_{m}^{1} \bigr) \\ &\qquad{}\oplus F_{i} \bigl(T_{i1}x_{1}^{2}, \ldots,T_{ii-1}x_{i-1}^{2},T_{ii}x_{i}^{1},T_{ii+1}x_{i+1}^{1}, \ldots,T_{im}x_{m}^{1} \bigr) \bigr\Vert \\ &\qquad{}+ \bigl\Vert F_{i} \bigl(T_{i1}x_{1}^{2}, \ldots,T_{ii-1}x_{i-1}^{2},T_{ii}x_{i}^{2},T_{ii+1}x_{i+1}^{1}, \ldots,T_{im}x_{m}^{1} \bigr) \\ &\qquad{}\oplus F_{i} \bigl(T_{i1}x_{1}^{2}, \ldots,T_{ii-1}x_{i-1}^{2},T_{ii}x_{i}^{2},T_{ii+1}x_{i+1}^{2}, \ldots,T_{im}x_{m}^{1} \bigr) \bigr\Vert +\cdots \\ &\qquad{}+ \bigl\Vert F_{i} \bigl(T_{i1}x_{1}^{2}, \ldots,T_{ii-1}x_{i-1}^{2},T_{ii}x_{i}^{2},T_{ii+1}x_{i+1}^{2}, \ldots,T_{im}x_{m}^{1} \bigr) \\ &\qquad{}\oplus F_{i} \bigl(T_{i1}x_{1}^{2}, \ldots,T_{ii-1}x_{i-1}^{2},T_{ii}x_{i}^{2},T_{ii+1}x_{i+1}^{2}, \ldots,T_{im}x_{m}^{2} \bigr) \bigr\Vert \\ &\quad \leq\lambda_{F_{i1}} \bigl\Vert T_{i1}x_{1}^{1} \oplus T_{i1}x_{1}^{2} \bigr\Vert + \lambda_{F_{i2}} \bigl\Vert T_{i2}x_{2}^{1} \oplus T_{i2}x_{2}^{2} \bigr\Vert +\cdots \\ &\qquad{}+\lambda_{F_{ii-1}} \bigl\Vert T_{ii-1}x_{i-1}^{1} \oplus T_{ii-1}x_{i-1}^{2} \bigr\Vert + \lambda_{F_{ii}} \bigl\Vert T_{ii}x_{i}^{1} \oplus T_{ii}x_{i}^{2} \bigr\Vert \\ &\qquad{}+\lambda_{F_{ii+1}} \bigl\Vert T_{ii+1}x_{i+1}^{1} \oplus T_{ii+1}x_{i+1}^{2} \bigr\Vert +\cdots+ \lambda_{F_{im}} \bigl\Vert T_{im}x_{m}^{1} \oplus T_{im}x_{m}^{2} \bigr\Vert . \end{aligned}$$

By using the Lipschitz continuity of $T_{ij}$-operator in Eq. (19), we have

20 $$\begin{aligned} & \bigl\Vert F_{i} \bigl(T_{i1}x_{1}^{1}, \ldots,T_{ii-1}x_{i-1}^{1},T_{ii}x_{i}^{1},T_{ii+1}x_{i+1}^{1}, \ldots,T_{im}x_{m}^{1} \bigr) \\ &\qquad {}\oplus F_{i} \bigl(T_{i1}x_{1}^{2},\ldots, T_{ii-1}x_{i-1}^{2},T_{ii}x_{i}^{2},T_{ii+1}x_{i+1}^{2}, \ldots,T_{im}x_{m}^{2} \bigr) \bigr\Vert \\ &\quad \leq\sum_{i \neq j, j=1}^{m} \lambda_{F_{ij}} \gamma_{ij} \bigl\Vert x_{j}^{1}\oplus x_{j}^{2} \bigr\Vert . \end{aligned}$$

Using Eq. (20) in Eq. (18) and then use it in Eq. (17), we have

21 $$\begin{aligned} \bigl\Vert y_{i}^{1} \oplus y_{i}^{2} \bigr\Vert \leq& \bigl\Vert y_{i}^{1}-y_{i}^{2} \bigr\Vert \\ \leq& \lambda_{N_{C}} \bigl(\alpha_{1}^{i}+ \lambda_{g_{i}}\alpha_{2}^{i} \bigr) \bigl\Vert x_{i}^{1}-x_{i}^{2} \bigr\Vert \\ &{}+ \lambda_{N_{C}} \Biggl[L_{i}(\lambda_{g_{i}}+ \lambda_{A_{i}}\lambda_{g_{i}}) \bigl\Vert x_{i}^{1}-x_{i}^{2} \bigr\Vert +L_{i}\rho_{i}\sum_{i\neq j, j=1}^{m} \lambda_{F_{ij}} \gamma_{ij} \bigl\Vert x_{j}^{1}-x_{j}^{2} \bigr\Vert \Biggr]. \end{aligned}$$

Now, Eq. (16) can be rewritten as

22 $$\begin{aligned} &\bigl\Vert Q \bigl(x_{1}^{1},x_{2}^{1}, \ldots,x_{m}^{1} \bigr)-Q \bigl(x_{1}^{2},x_{2}^{2}, \ldots,x_{m}^{2} \bigr) \bigr\Vert _{\ast} \\ &\quad \leq\sum _{i=1}^{m} \bigl\Vert y_{i}^{1}-y_{i}^{2} \bigr\Vert \\ &\quad \leq\sum_{i=1}^{m} \Biggl\{ \lambda_{N_{C}} \bigl[ \bigl(\alpha_{1}^{i}+ \lambda_{g_{i}}\alpha_{2}^{i} \bigr)+L_{i}( \lambda_{g_{i}}+\lambda_{A_{i}}\lambda_{g_{i}}) \bigr] \bigl\Vert x_{i}^{1}-x_{i}^{2} \bigr\Vert \\ &\qquad {} +\lambda_{N_{C}}L_{i}\rho_{i}\sum _{i\neq j, j=1}^{m}\lambda_{F_{ij}} \gamma_{ij} \bigl\Vert x_{j}^{1}-x_{j}^{2} \bigr\Vert \Biggr\} \\ &\quad \leq\sum_{i=1}^{m} \bigl\{ \lambda_{N_{C}} \bigl[ \bigl(\alpha_{1}^{i}+ \lambda_{g_{i}}\alpha_{2}^{i} \bigr)+L_{i}( \lambda_{g_{i}}+\lambda_{A_{i}}\lambda_{g_{i}}) \bigr] \bigr\} \bigl\Vert |x_{i}^{1}-x_{i}^{2} \bigr\Vert \\ &\qquad {}+\sum_{i=1}^{m}\lambda_{N_{C}}L_{i} \rho_{i}\sum_{i\neq j, j=1}^{m} \lambda_{F_{ij}} \gamma_{ij} \bigl\Vert x_{j}^{1}-x_{j}^{2} \bigr\Vert \\ &\quad \leq\sum_{j=1}^{m} \Biggl[ \lambda_{N_{C}} \bigl\{ \bigl(\alpha_{1}^{j}+ \alpha_{2}^{j}\lambda_{g_{i}} \bigr)+L_{j}( \lambda_{g_{j}}+\lambda_{A_{j}}\lambda_{g_{j}}) \bigr\} \\ &\qquad{}+ \lambda_{N_{C}} \sum_{i\neq j, i=1}^{m} L_{i}\rho_{i}\lambda_{F_{ij}}\gamma_{ij} \Biggr] \bigl\Vert x_{j}^{1}-x_{j}^{2} \bigr\Vert \\ &\quad = \sum_{j=1}^{m} \theta_{j} \bigl\Vert x_{j}^{1}-x_{j}^{2} \bigr\Vert \\ &\quad \leq\theta\sum_{j=1}^{m} \bigl\Vert x_{j}^{1}-x_{j}^{2} \bigr\Vert \\ &\quad \leq\theta \bigl\Vert \bigl(x_{1}^{1},x_{2}^{1}, \ldots,x_{m}^{1} \bigr)- \bigl(x_{1}^{2},x_{2}^{2}, \ldots,x_{m}^{2} \bigr) \bigr\Vert _{\ast}, \end{aligned}$$

where $\theta= \max_{1\leq j \leq m}\theta_{j}$. Finally, from Eq. (22), Eq. (16) can be written as

23 $$\begin{aligned} \bigl\Vert Q \bigl(x_{1}^{1},x_{2}^{1}, \ldots,x_{m}^{1} \bigr)-Q \bigl(x_{1}^{2},x_{2}^{2}, \ldots,x_{m}^{2} \bigr) \bigr\Vert _{\ast}& \leq \theta\sum_{j=1}^{m} \bigl\Vert x_{j}^{1}-x_{j}^{2} \bigr\Vert \\ &= \theta \bigl\Vert \bigl(x_{1}^{1},x_{2}^{1}, \ldots,x_{m}^{1} \bigr)- \bigl(x_{1}^{2},x_{2}^{2}, \ldots,x_{m}^{2} \bigr) \bigr\Vert _{\ast}. \end{aligned}$$

It follows from the condition (9) that $0 < \theta< 1$. This implies that $Q: \mathcal{E}_{1}\times\mathcal{E}_{2}\times\cdots \times\mathcal{E}_{m} \rightarrow\mathcal{E}_{1}\times\mathcal {E}_{2}\times\cdots\times\mathcal{E}_{m}$ is a contraction which in turn implies that there exists a unique $(x_{1}^{\ast},x_{2}^{\ast},\ldots, x_{m}^{\ast}) \in\mathcal {E}_{1}\times\mathcal{E}_{2}\times\cdots\times\mathcal{E}_{m}$ such that $Q(x_{1}^{\ast},x_{2}^{\ast},\ldots, x_{m}^{\ast})=(x_{1}^{\ast },x_{2}^{\ast},\ldots, x_{m}^{\ast})$. Thus, $(x_{1}^{\ast},x_{2}^{\ast },\ldots, x_{m}^{\ast})$ is the unique solution of problem (3). Now, we prove that $x_{i}^{n}\rightarrow x_{i}^{\ast}$ as $n \rightarrow\infty$ for $i=1,2,\ldots, m$. In fact, it follows from Eq. (8) and the Lipschitz continuity of the relaxed resolvent operator that

24 $$\begin{aligned} \bigl\Vert x_{i}^{n+1} \oplus x_{i}^{\ast} \bigr\Vert ={}& \bigl\Vert \bigl[x_{i}^{n}-g_{i} \bigl(x_{i}^{n} \bigr) + J_{\lambda_{i}, M_{i}}^{I_{i}-A_{i}} \bigl[(I_{i}-A_{i}) \bigl(g_{i} \bigl(x_{i}^{n} \bigr) \bigr) \\ &{}+\rho_{i}F_{i} \bigl(T_{i1}x_{1}^{n},T_{i2}x_{2}^{n}, \ldots,T_{im}x_{m}^{n} \bigr) \bigr] +w_{i}^{n}\oplus \bigl[x_{i}^{\ast}-g_{i} \bigl(x_{i}^{\ast} \bigr) \\ &{}+J_{\lambda_{i}, M_{i}}^{I_{i}-A_{i}} \bigl[(I_{i}-A_{i}) \bigl(g_{i} \bigl(x_{i}^{\ast} \bigr) \bigr) \\ &{}+\rho_{i}F_{i} \bigl(T_{i1}x_{1}^{\ast},T_{i2}x_{2}^{\ast}, \ldots,T_{im}x_{m}^{\ast} \bigr) \bigr] \bigr] \bigr] \bigr\Vert \\ \leq {}&\bigl\Vert \bigl(x_{i}^{n}-g_{i} \bigl(x_{i}^{n} \bigr) \bigr)\oplus \bigl(x_{i}^{\ast}-g_{i} \bigl(x_{i}^{\ast} \bigr) \bigr) \bigr\Vert \\ &{}+ \bigl\Vert J_{\lambda_{i}, M_{i}}^{I_{i}-A_{i}} \bigl[(I_{i}-A_{i}) \bigl(g_{i} \bigl(x_{i}^{n} \bigr) \bigr) \\ &{}+\rho_{i}F_{i} \bigl(T_{i1}x_{1}^{n},T_{i2}x_{2}^{n}, \ldots,T_{im}x_{m}^{n} \bigr) \bigr] \\ &{}\oplus J_{\lambda_{i}, M_{i}}^{I_{i}-A_{i}} \bigl[(I_{i}-A_{i}) \bigl(g_{i} \bigl(x_{i}^{\ast} \bigr) \bigr) \\ &{}+ \rho_{i}F_{i} \bigl(T_{i1}x_{1}^{\ast},T_{i2}x_{2}^{\ast}, \ldots,T_{im}x_{m}^{\ast} \bigr) \bigr] \bigr\Vert + \bigl\Vert w_{i}^{n}\oplus0 \bigr\Vert . \end{aligned}$$

From the previous calculations, we have

25 $$\begin{aligned} \sum_{i=1}^{m} \bigl\Vert x_{i}^{n+1} \oplus x_{i}^{\ast} \bigr\Vert ={}& \sum_{i=1}^{m} \bigl\Vert x_{i}^{n+1} - x_{i}^{\ast} \bigr\Vert \\ \leq{} &\Biggl[\lambda_{N_{C}} \bigl\{ \bigl(\alpha_{1}^{j}+ \alpha_{2}^{j}\lambda_{g_{i}} \bigr)+L_{j}( \lambda_{g_{j}}+\lambda_{A_{j}}\lambda_{g_{j}}) \bigr\} \\ &{}+ \lambda_{N_{C}} \sum_{i\neq j, i=1}^{m} L_{i}\rho_{i}\lambda_{F_{ij}}\gamma_{ij} \Biggr]\sum_{j=1}^{m} \bigl\Vert x_{j}^{n} - x_{j}^{\ast} \bigr\Vert + \sum_{j=1}^{m} \bigl\Vert w_{j}^{n} \bigr\Vert \\ ={}& \sum_{j=1}^{m} \theta_{j} \bigl\Vert x_{j}^{n} - x_{j}^{\ast} \bigr\Vert +\sum_{j=1}^{m} \bigl\Vert w_{j}^{n} \bigr\Vert , \end{aligned}$$

where $a_{n}=\sum_{j=1}^{m}\|x_{j}^{n} - x_{j}^{\ast}\|$, $b_{n}=\sum_{j=1}^{m}\|w_{j}^{n}\|$. Algorithm 2 yields $\lim_{n\rightarrow\infty}b_{n}=0$. Now, Lemma 2.2 implies that $\lim_{n\rightarrow\infty}a_{n}=0$, and so $x_{j}^{n} \rightarrow x_{j}^{\ast}$ as $n\rightarrow\infty$ for $j=1,2,\ldots,m$. This completes the proof. □

## Conclusion

Two of the most troublesome and imperative issues identified with inclusions are the foundation of generalized inclusions and the improvement of an iterative calculation. In this article, two systems of variational inclusions were presented and contemplated, which is a broader aim than the numerous current systems of generalized ordered variational inclusions in the literature. An iterative calculation is performed with a weak ARD mapping to an inexact solution of our systems, and the convergence criterion is likewise addressed.

We comment that our outcomes are new and valuable for additionally investigations. Considerably more work is required in every one of these regions to address utilizations of the system of general ordered variational inclusions in engineering and physical sciences.
